# A concise, health service coverage index for monitoring progress towards universal health coverage

**DOI:** 10.1186/s12913-015-0859-3

**Published:** 2015-06-12

**Authors:** Anthony Leegwater, Wendy Wong, Carlos Avila

**Affiliations:** Abt Associates, 4550 Montgomery Ave, Suite 800 North, Bethesda, MD 20814 USA; University of Chicago (formerly Analyst at Abt Associates), Chicago, USA

**Keywords:** Health service coverage, Universal health coverage, Index, Principal component analysis

## Abstract

**Background:**

There is a growing international commitment to universal health coverage (UHC), but limited means to determine progress towards that goal. We developed a practical index for capturing health service coverage – a critical dimension of UHC -- that was more inclusive than previous methods.

**Methods:**

Our data included publicly-available, indicators reflecting health service delivery, infrastructure, human resources, and health expenditures for 103 countries. We selected a set of internally-consistent indicators and performed principal component analysis. Multiple imputation was used to address missing values. We extracted and rotated four components related to health service coverage and developed a composite index for each country for 2009.

**Results:**

Explaining cumulatively almost 80% of the total variance, the four extracted components were characterized as: 1) provision of services, 2) infrastructure and human resources, 3) immunization (provision of services), and 4) financial resources. The health service coverage index developed from these components demonstrated strong correlation with health outcome measures such as infant mortality and life expectancy, supporting its validity. Index values also appeared generally consistent with published reports and the regional distribution of health coverage.

**Conclusions:**

Our approach moved beyond common indicators of service coverage focused on infectious diseases and maternal and child health, to include information on necessary health inputs. The resulting, balanced, composite index of health service coverage demonstrated promise as a metric, likely to discriminate coverage levels between countries and regions. An important number of service provision indicators were correlated, therefore a reduced set of services performed well as a proxy for the full set of available indicators. This parsimonious index is a start toward simplifying the task of policy-makers monitoring progress on a key domain of universal health coverage.

**Electronic supplementary material:**

The online version of this article (doi:10.1186/s12913-015-0859-3) contains supplementary material, which is available to authorized users.

## Background

A growing share of countries across the globe are declaring a commitment to pursuing Universal Health Coverage (UHC) and introducing policies and approaches to advance toward that goal. International donors and multilateral organizations are supporting these initiatives, with UHC under serious consideration for the post-2015 development agenda [1]. Such attention raises the issue of the most appropriate metrics for progress towards universal health coverage. Individual indicators currently used to capture aspects of UHC are myriad. There is however no existing measure that captures multiple dimensions of UHC. Even the composite indicators that have been developed in this area are focused on service coverage. Such composite measures are limited in how they are constructed and what health services are covered. Therefore, existing approaches do not appear to meet the current and future needs of policy makers, who need concise metrics to monitor whether countries are advancing in covering their health needs.

The 2010 World Health Report provides the most commonly-referenced definition of universal health coverage, describing it as a goal where all people have access to health services when needed and avoid financial hardship in paying for those services [2]. This influential report features a conceptual framework with three dimensions of UHC: service coverage, financial coverage, and population coverage. Although a persuasive conceptual framework, additional effort is needed to operationalize measurable indicators for tracking coverage in practice, especially as each dimension has its own measurement complexities [3]. We focus here on the service coverage dimension as a critical element, while acknowledging that the other dimensions of UHC are also important but data constrained.

Health service coverage has traditionally been measured by type of disease and type of treatment. Given the profusion of disease conditions and treatments, there have been some efforts to create composite indicators. For example, Millennium Development Goals (MDG) Countdown Research Group constructed composite indices by compiling a selection of service coverage indicators representing various strengths or intervention areas of the maternal and child health (MCH) service delivery system [4, 5]. However, this approach was limited not only by focusing on maternal and child health services but also by giving the same, arbitrary, equal weight to each indicator. Save the Children recently published a Health Access Index that ranked 75 countries with high maternal and child mortality according to health services access [6]. Their approach included six indicators, including four that match with our approach, but also included a measure of equity and an outcome measure for newborn mortality. However, like the MDG Countdown group, the indicators were equally weighted across categories. In addition, the inclusion of an outcome measure in the index risked conflating a goal of improved health services with the means by which it could occur.

Other studies offered in-depth assessments of health coverage in a small group of countries that have instituted specific health insurance or social health protection schemes. Available indicators related to financial coverage (or risk protection) measure household out-of-pocket (OOP) spending and identify when households have exceeded certain levels of spending deemed catastrophic [7]. However, household consumption data require large, expensive survey efforts and are thus typically conducted only every five years or so in most developing countries. This leaves insufficient data at present to include catastrophic measures across countries in our analysis.

A key consideration in measuring progress towards UHC practically is the availability and use of existing data from current systems, to avoid duplicating monitoring systems and imposing additional reporting burden on countries. Current indicators of service provision are dominated by maternal, child and infectious diseases, leaving many other services under-represented. Adding complementary factors – indicators such as infrastructure, human resources, and financial resources -- in the production of overall health services to service provision indicators is a step toward alleviating MCH over-representation in prior estimates. Indeed, our view is that the coverage of services should respond to the health needs of the broad population, the services must be physically available, and financial resources should be available to prevent financial risk when using health care services. These elements should therefore be included to improve the measurement of service coverage.

The objective of this study was to develop a practical index to monitor countries as they expand health coverage by using widely available information from domestic and international sources. Our specific focus here given the data available is service coverage, developing a measure that overcomes some of the shortcomings of other measures and doing so for a large group of countries.

## Methods

Our data included indicators reflecting health service delivery, health infrastructure, human resources for health and health expenditures for 2000 to 2010 from publicly-available global databases. The data sources included World Development Indicators and the World Health Organization’s Global Health Observatory. Data analysis was conducted using Stata 12 unless otherwise noted.

We excluded indicators with more than 85% missing data and dropped country observations with more than 50% missing values over that time period, resulting in a database with 19 indicators for 103 countries. For the years 2000 to 2010, 41% of all indicator values were missing. However, policy makers and other interested parties would likely prefer an approach that is focused on a recent year for potential use for benchmarking. Thus, we selected 2009 as a recent year with a more reasonable level of missing values (Additional file 1: Table S1).

**Fig. 1:**
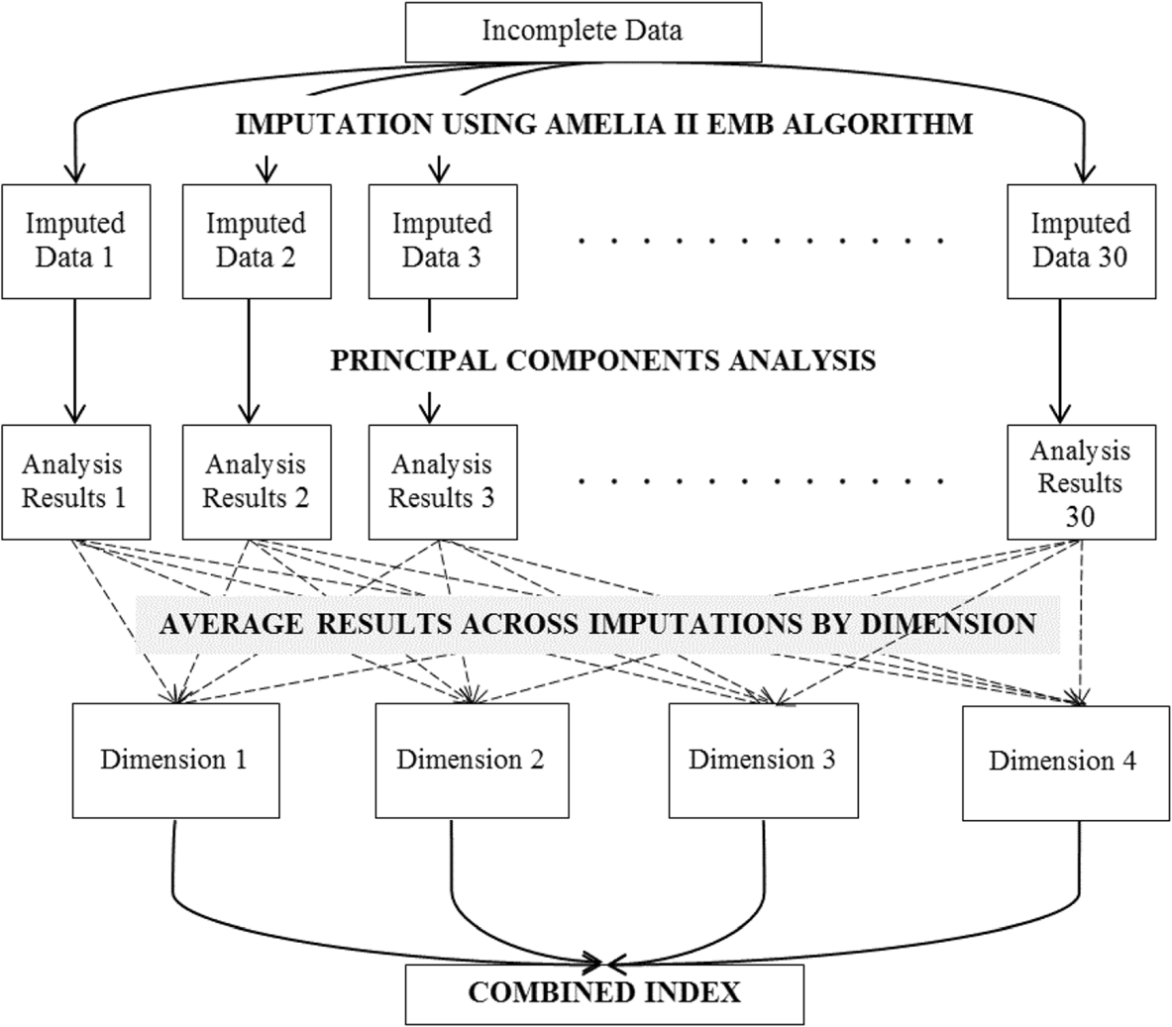
Overview of missing imputation and analysis process. (Source: authors. Description of missing value imputation and analysis process)

We imputed for missing values in 2009 using the broader 2000–2010 dataset (Fig. 1) utilizing a multiple imputation package with time series and cross-sectional capabilities [8]. As a result of this process, we had 30 complete data sets containing imputed values across 19 indicators and 103 countries (Additional file 1: Table S2.)

### Internal consistency

To explore relationships in the data and to examine whether they are suitable for such a principal components analysis approach, we examined correlations between the 19 potential indicators. We also calculated Cronbach coefficient alphas (c-alphas) for the entire group of indicators as well as those indicators that were initially grouped according to the three conceptual elements of health coverage under consideration: (1) infrastructure and human resources, (2) provision of services, and (3) financial resources for health. Each imputed data set was analyzed individually at this stage, but the reported values are means across the 30 imputed data sets. Based on the correlation and Cronbach alpha results, we removed three of the 19 indicators: antiretroviral therapy coverage, tuberculosis treatment, and health expenditures from sources other than household out-of-pocket payments (as share of public health expenditures). These indicators demonstrated low correlation with other potential service coverage indicators – and for tuberculosis an unexpected negative sign -- and contributed relatively little to a consistent set of indicators. We also removed the least correlated immunization variables – BCG, HepB3, and Pol3 -- from analysis to avoid dominating the overall measure with immunization-related variables. The remaining variables yielded c-alpha of 0 · 92 as a group, along with a Kaiser-Meyer-Olkin measure of greater than 0.8. We rejected the null hypothesis of non-intercorrelation using Bartlett’s sphericity test (Additional file 1: Table S3).

### Principal components analysis

With the remaining 13 indicators, we performed principal components analysis (PCA). In pursuing PCA, the following steps were necessary: extracted components, rotated components to ease interpretation and constructed weights from component loading. Our analytical approach therefore was to identify intermediate components or dimensions of service coverage as well as a final index representing overall health service coverage provision for the 103 countries.

The first four components extracted by PCA, with eigenvalues greater than one, were selected and then varimax rotation applied to assist interpretation (Table 1). From these loadings, we created weights based on the normalized square of the loading. Indicator weights constructed in this way represented the proportion of the indicator’s total unit variance explained by the component on which the indicator loaded.

**Table 1:** Average component loadings (Source: authors’ calculations. Loadings near 0.30 and higher bolded. Mean values across imputations)

	Comp 1	Comp 2	Comp 3	Comp 4
Antenatal care 1+ visits	0 · 25	0 · 03	0 · 09	0 · 16
Antenatal care 4+ visits	**0 · 35**	0 · 02	-0 · 01	0 · 08
Births attended by skilled staff	**0 · 31**	0 · 14	0 · 11	0 · 01
Contraceptive prevalence	0 · 24	0 · 09	0 · 18	-0 · 01
TB detection rate	**0 · 30**	0 · 03	0 · 09	-0 · 06
Immunization DTP3	-0 · 01	0 · 10	**0 · 48**	0 · 10
Immunization measles	-0 · 03	0 · 07	**0 · 50**	0 · 06
Hospital beds	0 · 12	**0 · 39**	0 · 03	0 · 03
Physicians	0 · 25	**0 · 34**	-0 · 03	-0 · 07
Nurses	0 · 09	**0 · 41**	0 · 01	0 · 01
Health expenditure, public	-0 · 01	0 · 07	0 · 14	**0 · 50**
Health expenditure, per capita	**0 · 35**	0 · 12	-0 · 09	0 · 08
Health expenditure, not OOP	-0 · 01	0 · 01	0 · 09	**0 · 54**
Explained variance	3 · 24	2 · 70	2 · 25	2 · 02
Explained/total variance	0 · 32	0 · 26	0 · 22	0 · 20

### Scores by component

When multiplied by the indicator values, the weights produced scores for each of the four components. These scores could be considered intermediate measures of service coverage. We took the mean of these intermediate scores across the data sets [9, 10]. Then, we rescaled the intermediate scores, relative to the minimum value for that score among the 103 countries and an ‘ideal’ value based on a hypothetical country with strong, health service coverage. This country was defined as having services covered for 100% of the relevant population and values for the other indicators set at the median of high-income countries. Values closer to 0 for that intermediate score indicated a country was near the bottom of the group in that dimension, while values closer to 100 approached a ‘target’ level of health coverage. We combined these intermediate scores into a final, overall coverage index by weighting each component by its share of the explained variance [8, 11] and summing the products (see Table 2).

**Table 2:** Countries included in analysis and their calculated overall service coverage scores (source: authors’ calculations)

Afghanistan	5 4	Gambia	54 · 1	Nicaragua	70 · 6
Albania	72 · 3	Georgia	70 · 6	Niger	21 · 4
Algeria	75 · 3	Ghana	54 · 7	Nigeria	16 · 8
Argentina	98 · 6	Guinea	1 · 8	Pakistan	39 · 2
Armenia	79 · 0	Guinea-Bissau	29 · 7	Panama	77 · 4
Azerbaijan	68 · 6	Guyana	75 · 8	Paraguay	60 · 5
Bangladesh	32 · 3	Honduras	67 · 4	Peru	71 · 2
Belarus	119 · 6	India	31 · 4	Philippines	45 · 4
Belize	70 · 4	Indonesia	53 · 2	Romania	95 · 3
Benin	38 · 3	Iran	73 · 1	Russia	96 · 4
Bhutan	68 · 1	Iraq	51 · 1	Rwanda	56 · 4
Bolivia	57 · 2	Jamaica	70 · 7	Sao Tome and Principe	53 · 4
Bosnia and Herz.	74 · 6	Jordan	85 · 2	Senegal	38 · 7
Botswana	81 · 5	Kazakhstan	98 · 1	Sierra Leone	26 · 3
Brazil	85 · 7	Kenya	46 · 9	South Africa	61 · 8
Bulgaria	94 · 4	Kyrgyzstan	77 · 0	Sri Lanka	68 · 2
Burkina Faso	47 · 3	Laos	20 · 4	Sudan	29 · 5
Burundi	48 · 3	Latvia	94 · 4	Swaziland	67 · 3
Cambodia	47 · 9	Lesotho	60 · 3	Syria	56 · 0
Cameroon	37 · 5	Lithuania	108 · 3	Tajikistan	52 · 7
Chile	77 · 2	Macedonia	88 · 1	Tanzania	54 · 9
China	75 · 3	Madagascar	36 · 7	Thailand	76 · 4
Colombia	78 · 5	Malawi	58 · 4	Togo	35 · 5
Congo, Dem. Rep.	40 · 4	Malaysia	72 · 1	Tonga	80 · 1
Costa Rica	74 · 5	Maldives	82 · 9	Tunisia	76 · 9
Cote d’Ivoire	26 · 6	Mali	28 · 9	Turkey	80 · 8
Cuba	122 · 4	Mexico	77 · 1	Turkmenistan	81 · 4
Djibouti	60 · 8	Moldova	83 · 7	Uganda	20 · 8
Dominican Republic	54 · 9	Mongolia	82 · 8	Ukraine	91 · 5
Ecuador	64 · 5	Morocco	58 · 5	Uruguay	93 · 4
Egypt	64 · 1	Mozambique	43 · 6	Uzbekistan	82 · 1
El Salvador	73 · 3	Myanmar	32 · 0	Vietnam	59 · 5
Eritrea	51 · 0	Namibia	66 · 9	Yemen	30 · 3
Ethiopia	30 · 9	Nepal	48 · 9	Zambia	51 · 8
Fiji	76 · 9				

The first four selected components explained cumulatively 79% of the variance. Upon rotation for easier interpretation, the average loadings and patterns were found.

We should note that our data are at the country level, based on secondary data sources, and do not include human subjects, human material, or human data.

## Results

Interpretation of the principal components appeared to be relatively straightforward, as we identified dimensions related to the provision of services, infrastructure and human resources, and financial resources for health. Provision-of-services variables loaded most heavily on Component 1, although health expenditure per capita also loaded on this component. Component 2 related to health sector infrastructure and human resources, with hospital beds, physicians, and nurses loaded on it. Indicators related to immunizations – a key part of health service coverage – loaded most heavily on Component 3. Finally, Component 4 related to financial resources for health, as evidenced by the loadings for public health expenditures and health expenditures from sources other than out-of-pocket payments by households. The lack of strong average loadings (>0 · 30) on two of the indicators (one or more antenatal visits and contraceptive prevalence) reflected some flipping in the component loadings across imputed data sets, as service provision indicators loaded more heavily on Components 2 and 3 in some of these imputations. On the whole, however, the results were generally consistent with a structure in which the provision of services, infrastructure and human resources, and financial resources for health indicators represented different dimensions of health service coverage.

To check the face validity of our health service coverage index, we compared the index with three external measures: (1) surrogates of health coverage such as infant mortality and life expectancy, (2) published reports of health coverage and (3) a map of the regional distribution of health coverage. In general, the service coverage index demonstrated a statistically significant correlation with surrogates of health coverage, was consistent with health coverage levels from individual country reports while presenting broader, more useful information, and offered a reasonable depiction of the regional distribution of health coverage.

**Fig. 2:**
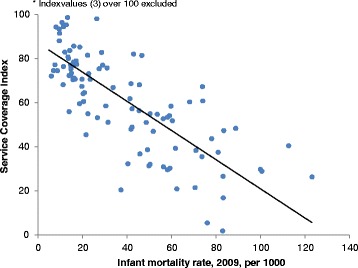
Relation between service coverage index and infant mortality. (Source: authors’ calculations for index and infant mortality data from World Bank’s World Development Indicators. Infant mortality rate in 2009, expressed per thousand. Three service coverage index values over 100 -for Belarus, Cuba, and Lithuania- were excluded

We expected countries with lower levels of service coverage to have higher levels of infant mortality. This proved to be the case (Fig. 2) with infant mortality strongly associated with our derived service coverage index (Spearman’s rho = -0 · 81, p < 0 · 001). One would also generally expect that countries with higher service coverage would exhibit longer life expectancy. Again, this was indeed the case when comparing the service coverage index with total life expectancy in 2009 (Spearman’s rho = 0 · 68, p < 0 · 001). Other important measures of health – under-five mortality and life expectancy – exhibit similar associations with the constructed index.

There were also benefits from looking at the four components that contribute to the overall service coverage index. These components provided detail otherwise obscured by the overall index, such as whether a country was lagging behind in one or more aspects of health service coverage or instead had broad-based support for its service coverage. Table 2 provides the values of the individual components, as well as the overall coverage value, for 11 countries. We discuss these values in more detail in the discussion section.

**Fig. 3:**
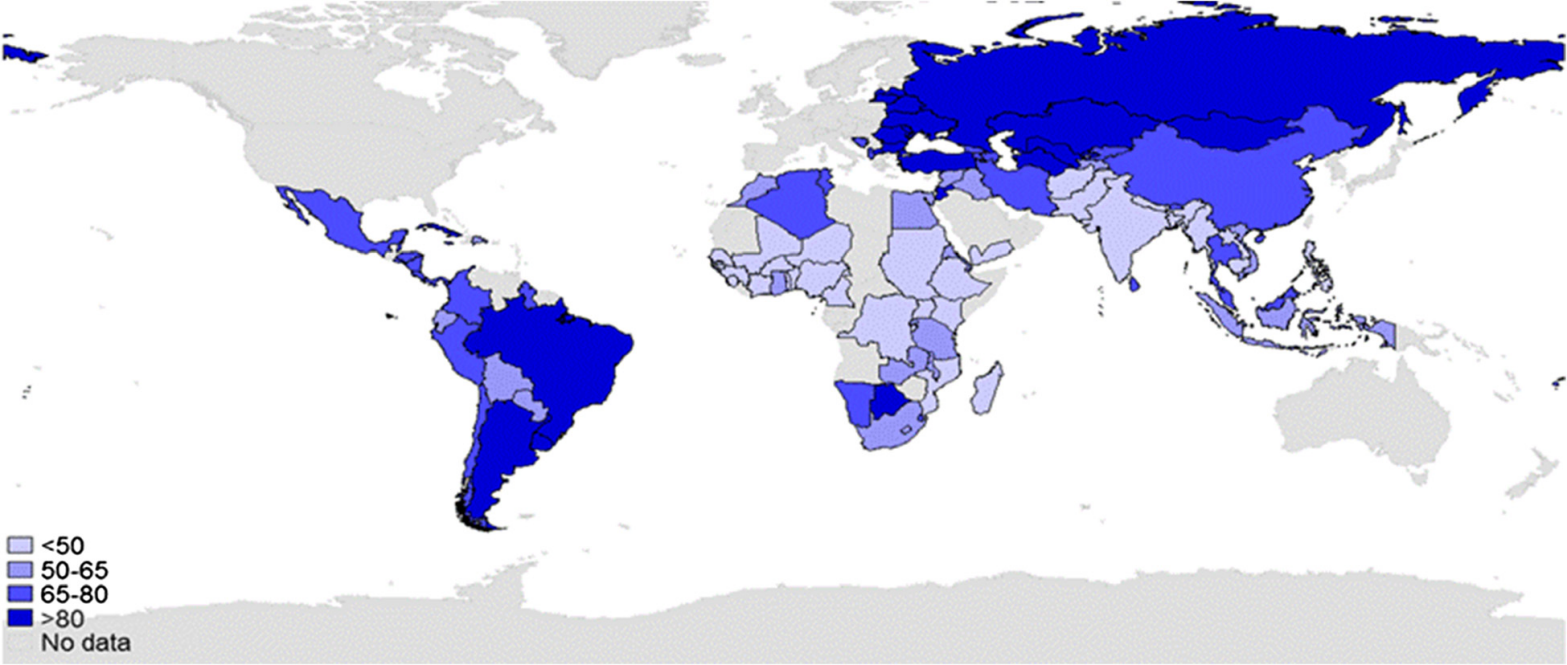
Health service coverage index for 103 countries. (Source: global map of service coverage index calculated by authors)

Figure 3 provides a map of our service coverage index. The colors distinguished where a country was in the quintiles of the service coverage index (from light to dark blue, gray represents no data). Africa was the region with the lowest levels of service coverage, with most countries with available data in the bottom or second–lowest quintile for health coverage. Eastern Europe and Asia displayed the highest concentration of coverage with nearly all countries in the highest or second highest quintiles.

## Discussion

Our objective was to develop an index of health service coverage that would help policymakers confront the myriad indicators available of relevance to monitoring coverage of health services. This index offered a concise set of indicators that were practical, balanced, and valid measures of service coverage across a wide swath of low- and lower-middle income countries. From a large list of potential indicators collated from existing information -- rather than requiring new data collection -- we selected a limited set of 16 variables covering three major domains: infrastructure and human resources, provision of services, and financial resources for health. This set of indicators moved beyond typical indicators of service coverage in use, which focused on infectious diseases and maternal and child health, to include information on the necessary health inputs of infrastructure, human resources, and financing resources. This resulted in a more balanced, composite index of health service coverage.

This index displayed promising results as a valid measure when related to health outcomes. It demonstrated strong correlation with such measures, particularly with infant mortality and life expectancy. Though considerable uncertainty remains about the true level of health service coverage, and the comprehensiveness of services in countries may vary substantially, we concluded that our health coverage index demonstrated promise as a metric, as it was likely to discriminate coverage levels between countries and regions.

While at present no published measure captures fully the conceptual construct of universal health coverage proposed by the World Health Organization, our constructed index improved on existing approaches to measuring service coverage, an important element of UHC. An important number of service provision indicators were correlated; therefore, a reduced set of services could perform well as a proxy for the full set of available indicators. Also, the MDG indicators for reproductive, maternal, newborn, child and adolescent health, and infectious diseases tend to be over-represented in service coverage measures including existing composite indices. Thus, our set of indicators – including inputs such as human resources for health and health infrastructure -- provided a more balanced view of service coverage. Our approach has also advanced beyond existing composite index measures of coverage by using data-driven weights rather than fixed or arbitrary weights [12].

Other studies, rather than relying on large sets of indicators or composite measures, instead offered assessments of health service coverage or universal health coverage in particular countries or small groups of countries that have instituted specific health insurance or social health protection schemes. By their nature, these studies are limited in what they can say on a cross-national basis, whereas our approach encouraged such comparisons. In addition, these measures may miss important aspects of service coverage in these countries. Take for example Tanzania (Table 3). Based on its overall index score of 54 · 9, Tanzania was in middle of the 103 countries. However, Mills and colleagues [13] placed its level of health insurance at approximately 10% and therefore at the low end of countries pushing toward universal coverage. This result illustrates the difference between legal protection (insurance) and access to public and private health services outside of formal insurance schemes. Our analysis indicated that Tanzania boasted some strength in terms of financial resources and immunization coverage. For Rwanda, 92% are said to be enrolled in government insurance programs [14], but our overall service coverage index is 56 · 4, with a particularly low value for the service provision component. Similarly, South Africa was said to have more than 95% health coverage [15], but our index revealed a more complicated picture, with relatively poor standing in infrastructure and human resources and especially immunization.

**Table 3:** Derived service coverage components and index (source: authors’ calculations)

	1. Service provision	2. Infrastructure & human resources	3. Immunization	4. Financial resources	Overall index
Bangladesh	5 · 5	22 · 1	75 · 7	40 · 6	32 · 3
Philippines	42 · 5	26 · 6	65 · 2	53 · 1	45 · 4
Indonesia	55 · 9	33 · 3	61 · 0	66 · 9	53 · 2
Ghana	39 · 2	34 · 0	79 · 3	80 · 0	54 · 7
Tanzania	39 · 7	31 · 6	72 · 4	91 · 2	54 · 9
Rwanda	38 · 4	44 · 9	76 · 3	78 · 4	56 · 4
Vietnam	54 · 2	53 · 9	80 · 5	52 · 0	59 · 5
South Africa	82 · 7	44 · 8	37 · 4	78 · 0	61 · 8
Costa Rica	87 · 1	49 · 0	73 · 1	89 · 7	74 · 5
Thailand	67 · 4	51 · 9	95 · 6	102 · 1	76 · 4
Mexico	88 · 0	60 · 1	86 · 9	71 · 5	77 · 1

We acknowledge that our approach was constrained in certain respects. Certain key indicators, because of their lack of general availability or low frequency of collection, were missing from the service coverage index. Indicators of population coverage by socio-economic status were relatively sparse -- collected only every five years or so for most countries -- preventing a global look at the important issues of equity and household risk protection. Overall figures of provision of services may have obscured the fact that the poor were less likely to access services even from government programs. In addition, household expenditures data were also needed to get a view of catastrophic health expenditures. Such expenditures may persist despite growth in health coverage by certain measures [16]. Indicators measuring provision for chronic diseases were rarely reported, despite the increasing attention paid to these diseases in the public health arena. This paucity reflected inadequate monitoring and surveillance [17]. Another acknowledged limitation of our approach, as with most data sets including large groups of countries, was that our data contained a significant number of missing values. The imputations necessary to deal with these missing values created some differences in PCA results – which indicators loaded on what components and with what strength -- between data sets and have reduced the average loadings for certain indicators.

## Conclusions

Measuring progress towards UHC requires a standard set of indicators that allow comparisons at the regional and subnational level. Our approach was a starting point, an attempt to outline what data were available for monitoring UHC, how such available data – focused on a key element of UHC (service coverage) -- could be condensed into a parsimonious index, and to suggest what might be done to advance the measurement and tracking of service coverage in future research. Looking forward, other indicators can be incorporated into this approach such as service provision indicators of chronic diseases, cancer, injuries and health preventive services as such data become increasingly available. The strong global movement towards universal health coverage requires a reliable system able to monitor progress, government commitments, and donor expectations. Further, monitoring progress requires a limited set of valid and reliable indicators, avoiding additional burdening of the overstretched country information systems.

## Additional file

Additional file 1: Table S1. Potential Indicators of Universal Health Coverage (Sources: multiple as described in table. Asterisk denotes indicators that were redefined as 100-original value so that they are positively associated with coverage). **Table S2.** Summary statistics (Source: author’s calculations based on 30 imputed data sets for 103 countries). **Table S3.** Correlation Matrix (Source: author’s calculations based on average values from 30 imputed data sets).

## Abbreviations

DHS: Demographic and Health Survey
MCH: Maternal and Child Health
MDG: Millennium Development Goals
OOP: Out of pocket
PCA: Principal Components Analysis
UHC: Universal Health Coverage
UNAIDS: Joint United Nations Programme on HIV/AIDS
UNICEF: United Nations Children’s Fund
WDI: World Development Indicators (World Bank)
WHO: World Health Organization
