# Supplementary material for: A concise, health service coverage index for monitoring progress towards universal health coverage
Source: BMC Health Serv Res. 2015 Jun 12;15:230. doi: 10.1186/s12913-015-0859-3 (PMC4474431; doi:10.1186/s12913-015-0859-3)
Supplement: Additional file 1: Table S1. — Potential Indicators of Universal Health Coverage (Sources: multiple as described in table. Asterisk denotes indicators that were redefined as 100-original value so that they are positively associated with coverage). Table S2. Summary statistics (Source: author’s calculations based on 30 imputed data sets for 103 countries). Table S3. Correlation Matrix (Source: author’s calculations based on average values from 30 imputed data sets). [file 12913_2015_859_MOESM1_ESM.docx]

**Additional file 1**

**Table S1**. Potential Indicators of Universal Health Coverage (Sources: multiple as described in table. Asterisk denotes indicators that were redefined as 100-original value so that they are positively associated with coverage.)

| Indicator Name | Included? | Source |
| --- | --- | --- |
| Provision of Services |  |  |
| Antenatal care, 1+ visit (% of women) | Yes | WHO, DHS |
| Antenatal care, 4+ visits (% of women) | Yes | WHO, DHS |
| Antiretroviral therapy coverage (% of people with advanced HIV infection) | No, low correlation | WDI, WHO, UNAIDS |
| Acute respiratory infection treatment (% of children under 5 taken to a health provider) | No, high % of missing data | WDI, DHS |
| Births attended by skilled health staff (% of total births) | Yes | WDI, WHO, UNICEF |
| Children with fever receiving antimalarial drugs (% of children under age 5 with fever) | No, high % of missing data | WDI, WHO, DHS |
| Condom use with non-regular partner, female (% adults 15-49) | No, high % of missing data | WDI |
| Condom use with non-regular partner, male (% adults 15-49) | No, high % of missing data | WDI |
| Contraceptive prevalence among married women, any modern method (% of married women ages 15-49) | No | WDI, DHS, UN |
| Contraceptive prevalence, any method (% of married women ages 15-49) | Yes | WDI, DHS, UN |
| Diarrhea treatment (% of children under 5 receiving oral rehydration and continued feeding) | No, high % of missing data | WDI, DHS |
| Immunization, BCG (% of one-year-old children) | No, low correlation | WDI, UNICEF |
| Immunization, DTP3 (% of children ages 12-23 months) | Yes | WDI, UNICEF |
| Immunization, HepB3 (% of one-year-old children) | No, low correlation | WDI, UNICEF |
| Immunization, measles (% of children ages 12-23 months) | Yes | WDI, UNICEF |
| Immunization, Pol3 (% of one-year-old children) | No, low correlation | WDI, UNICEF |
| Inpatient admission rate (% of population ) | No, high % of missing data | WDI, UNICEF |
| Outpatient visits per capita | No, high % of missing data | WDI |
| Percentage of deliveries at a health facility | No, high % of missing data | DHS |
| Tuberculosis case detection rate, all forms (% of estimated incident cases) | Yes | WDI |
| Tuberculosis treatment success rate (% of registered cases) | No, low correlation and unexpected sign | WDI, WHO |
| Unmet need for contraception (% of married women ages 15-49) | No, high % of missing data | WDI, WHO, DHS |
| Use of insecticide-treated bed nets (% of under-5 population) | No, high % of missing data | WDI, WHO, DHS |
| Infrastructure and human resources |  |  |
| Community and Traditional Health Workers density (per 1,000 population) | No, high % of missing data | WHO |
| Hospital beds (per 1,000) | Yes | WDI |
| Laboratory Health Workers density (per 1,000) | No, high % of missing data | WHO |
| Midwives (per 1,000) | No, high % of missing data | WDI |
| Nurses (per 1,000) | Yes | WDI, WHO |
| Physicians (per 1,000) | Yes | WDI, WHO |
| Total density: District/rural hospitals (per 100,000 population) | No, high % of missing data | WHO |
| Total density: Health centers (per 100,000 population) | No, high % of missing data | WHO |
| Total density: Health posts (per 100,000 population) | No, high % of missing data | WHO |
| Total density: Provincial hospitals (per 100,000 population) | No, high % of missing data | WHO |
| Total density: Specialized hospitals (per 100,000 population) | No, high % of missing data | WHO |
| Financial Resources for Health |  |  |
| Health expenditure per capita | Yes | WDI, WHO |
| Health expenditure, public (% of total health expenditure) | Yes | WDI, WHO |
| Health expenditure that is not out-of-pocket (100 - % of private expenditure on health) | No, low correlation | WDI, WHO |
| Health expenditure that is not out-of-pocket (100 - % of total expenditure on health) | Yes | WDI, WHO |

**Table S2**. Summary statistics (Source: author’s calculations based on 30 imputed data sets for 103 countries)

|  | N | Mean | Std. Dev | Min | Max |
| --- | --- | --- | --- | --- | --- |
| Antenatal care 1+ visits | 3090 | 88.31 | 14.83 | 2.51 | 100.00 |
| Antenatal care 4+ visits | 3090 | 67.63 | 24.23 | 3.42 | 99.99 |
| Births attended by skilled staff | 3090 | 78.92 | 24.56 | 1.47 | 100.00 |
| Contraceptive prevalence | 3090 | 52.96 | 23.16 | 1.95 | 99.18 |
| TB detection rate | 3090 | 69.68 | 17.98 | 11.00 | 100.00 |
| Immunization DTP3 | 3090 | 88.85 | 10.08 | 57.00 | 99.00 |
| Immunization measles | 3090 | 87.54 | 12.05 | 51.00 | 99.00 |
| Hospital beds | 3090 | 2.32 | 2.25 | 0.00 | 17.14 |
| Physicians | 3090 | 1.29 | 1.35 | 0.00 | 10.68 |
| Nurses | 3090 | 2.45 | 2.78 | 0.00 | 35.49 |
| Health expenditure, public | 3090 | 51.07 | 18.34 | 8.19 | 92.72 |
| Health expenditure, per capita | 3090 | 20588.55 | 21533.18 | 1067.31 | 83561.78 |
| Health expenditure, not OOP | 3090 | 60.54 | 19.02 | 8.71 | 92.92 |

**Table S3**. Correlation Matrix (Source: author’s calculations based on average values from 30 imputed data sets)

|  | ANC1 | ANC2 | Births | Contrac | TB | DTP3 | Measles |
| --- | --- | --- | --- | --- | --- | --- | --- |
| Antenatal care 1+ visits | 1.00 |  |  |  |  |  |  |
| Antenatal care 4+ visits | 0.67 | 1.00 |  |  |  |  |  |
| Births attended by skilled staff | 0.65 | 0.60 | 1.00 |  |  |  |  |
| Contraceptive prevalence | 0.36 | 0.48 | 0.61 | 1.00 |  |  |  |
| TB detection rate | 0.36 | 0.35 | 0.49 | 0.45 | 1.00 |  |  |
| Immunization DTP3 | 0.46 | 0.37 | 0.51 | 0.50 | 0.37 | 1.00 |  |
| Immunization measles | 0.48 | 0.40 | 0.56 | 0.57 | 0.43 | 0.91 | 1.00 |
| Hospital beds | 0.32 | 0.23 | 0.53 | 0.39 | 0.29 | 0.32 | 0.39 |
| Physicians | 0.32 | 0.38 | 0.56 | 0.48 | 0.38 | 0.30 | 0.39 |
| Nurses | 0.25 | 0.24 | 0.44 | 0.32 | 0.20 | 0.29 | 0.34 |
| Health expenditure, public | 0.44 | 0.40 | 0.40 | 0.40 | 0.33 | 0.41 | 0.50 |
| Health expenditure, per capita | 0.37 | 0.49 | 0.55 | 0.53 | 0.46 | 0.28 | 0.36 |
| Health expenditure, not OOP | 0.48 | 0.38 | 0.34 | 0.32 | 0.26 | 0.32 | 0.41 |
|  | Beds | Physicians | Nurses | Hlth Exp. Publ. | Hlth Exp. Cap | Not OOP |  |
| Hospital beds | 1.00 |  |  |  |  |  |  |
| Physicians | 0.75 | 1.00 |  |  |  |  |  |
| Nurses | 0.79 | 0.76 | 1.00 |  |  |  |  |
| Health expenditure, public | 0.25 | 0.20 | 0.20 | 1.00 |  |  |  |
| Health expenditure, per capita | 0.42 | 0.54 | 0.35 | 0.41 | 1.00 |  |  |
| Health expenditure, not OOP | 0.14 | 0.08 | 0.09 | 0.90 | 0.39 | 1.00 |  |
